# *Halobacterium salinarum* virus *ChaoS9*, a Novel Halovirus Related to PhiH1 and PhiCh1

**DOI:** 10.3390/genes10030194

**Published:** 2019-03-01

**Authors:** Mike Dyall-Smith, Peter Palm, Gerhard Wanner, Angela Witte, Dieter Oesterhelt, Friedhelm Pfeiffer

**Affiliations:** 1 Computational Biology Group, Max-Planck-Institute of Biochemistry, Am Klopferspitz 18, 82152 Martinsried, Germany; mike.dyallsmith@gmail.com (M.D.-S.); hp.palm@kabelmail.de (P.P.); oesterhe@biochem.mpg.de (D.O.); 2 Veterinary Biosciences, Faculty of Veterinary and Agricultural Sciences, University of Melbourne, Parkville, VIC 3052, Australia; 3 AG Ultrastrukturforschung, Biozentrum der LMU, Großhadernerstrasse 2-4, 82152 Martinsried, Germany; wanner@lrz.uni-muenchen.de; 4 Department of Microbiology, Immunobiology and Genetics, MFPL Laboratories, University of Vienna, Dr. Bohr-Gasse 9, 1030 Vienna, Austria; angela.witte@univie.ac.at

**Keywords:** halovirus, caudovirus, halobacteria, *Archaea*, *haloarchaea*, genome inversion, transposon

## Abstract

The unexpected lysis of a large culture of *Halobacterium salinarum* strain S9 was found to be caused by a novel myovirus, designated ChaoS9. Virus purification from the culture lysate revealed a homogeneous population of caudovirus-like particles. The viral genome is linear, dsDNA that is partially redundant and circularly permuted, has a unit length of 55,145 nt, a G + C% of 65.3, and has 85 predicted coding sequences (CDS) and one tRNA (Arg) gene. The left arm of the genome (0–28 kbp) encodes proteins similar in sequence to those from known caudoviruses and was most similar to myohaloviruses phiCh1 (host: *Natrialba magadii*) and phiH1 (host: *Hbt. salinarum*). It carries a tail-fiber gene module similar to the invertible modules present in phiH1 and phiCh1. However, while the tail genes of ChaoS9 were similar to those of phiCh1 and phiH1, the Mcp of ChaoS9 was most similar (36% aa identity) to that of *Haloarcula hispanica* tailed virus 1 (HHTV-1). Provirus elements related to ChaoS9 showed most similarity to tail/assembly proteins but varied in their similarity with head/assembly proteins. The right arm (29–55 kbp) of ChaoS9 encoded proteins involved in DNA replication (ParA, RepH, and Orc1) but the other proteins showed little similarity to those from phiH1, phiCh1, or provirus elements, and most of them could not be assigned a function. ChaoS9 is probably best classified within the genus *Myohalovirus*, as it shares many characteristics with phiH1 (and phiCh1), including many similar proteins. However, the head/assembly gene region appears to have undergone a recombination event, and the inferred proteins are different to those of phiH1 and phiCh1, including the major capsid protein. This makes the taxonomic classification of ChaoS9 more ambiguous. We also report a revised genome sequence and annotation of *Natrialba* virus phiCh1.

## 1. Introduction

Viruses infecting extremely halophilic archaea (haloarchaea) include a variety of morphotypes, such as caudoviruses (e.g., phiH1), round viruses (e.g., SH1), pleomorphic viruses (e.g., His2), and spindle-shaped viruses (e.g., His1) [1,2]. In hypersaline environments such as salt lakes and saltern crystallizer ponds, the vast majority of prokaryotes are usually haloarchaea (Class Halobacteria) with cell concentrations reaching up to 10^8^ per mL, but the concentration of virus particles is often 10-fold higher [3], so that haloviruses are important regulators of host cell populations as well as major drivers of their evolution. Among the haloarchaeal caudoviruses, examples of myovirus-like and siphovirus-like viruses have been described, and both temperate and virulent (lytic) isolates have been reported [4,5,6,7]. With many haloarchaeal genome sequences now available, it is clear that proviruses are common and widespread, and can be present either as plasmids [8] or integrated into the host chromosome [9,10]. The high numbers of viruses in environmental samples, their stability, and the need for large quantities of salt for cultivation of haloarchaea, is a potential hazard for large scale culture of haloarchaea for biotechnological purposes, as virus contamination from medium components or the local environment could result in lysis of the cells.

For many decades, the biological function of bacteriorhodopsin was a focus of study in the Oesterhelt department of the Max Planck Institute (MPI) in Martinsried [11,12,13]. The bacteriorhodopsin producer strain *Halobacterium salinarum* strain S9 was derived from strain R1 (DSM 671). The R1 strain and derivative strains such as S9 have been widely used to produce commercial quantities of bacteriorhodopsin [14]. *Hbt. salinarum* S9 (originally strain R1S9) was first described in a 1979 review by Stoeckenius et al. [15] as a purple membrane overproducer strain derived by Lily Jan (unpublished) from strain R1 by nitrosoguanidine mutagenesis. It has been used in numerous studies, such as gene regulation and expression [16,17,18,19], and cell chemotaxis and phototaxis [20,21]. In some studies, this strain has also been labelled as *bat*+ [18]. 

Commercial production of pure bacteriorhodopsin entails regular, large-scale cultivation, a practice known to increase the likelihood of virus contamination that can lead to sudden lysis of the microbial cells in a bioreactor [22]. Such an event occurred in 2007 in a 1 m^3^ culture of *Hbt. salinarum* S9 being grown at the MPI laboratory. A similar, spontaneous lysis event had occurred in 1974 in the Oesterhelt group and a sample of culture fluid, which had been collected by Hartmut Michel, turned out to contain halovirus phiH [23]. Accordingly, it was initially assumed that phiH was also responsible for the 2007 event.

The aim of this study was to characterize the virus that caused the large-scale lysis of *Hbt. salinarum* S9 in 2007. This turned out to be a novel halovirus, related to phiH1 and phiCh1, which we named ChaoS9 (Chao: Caudovirus of haloarchaeal origin; S9: the affected strain). The virus morphology, proteins, and genome sequence were analyzed and compared with other described haloviruses and provirus elements in order to assess its novelty, evolution, and taxonomic position. During this study, we also resequenced Natrialba virus phiCh1.

## 2. Materials and Methods

### 2.1. Host Strain, Virus Isolation, Cultivation, and Purification

*Hbt. salinarum* S9 is a purple-membrane (Pum) constitutive strain [15]. It was grown aerobically in peptone/salts medium, as previously described [24]. Virus purification and DNA extraction followed the methods previously described for halovirus phiH [23]. Briefly, this involved filtration of the lysate through diatomaceous earth (DE), concentration of viral particles from the filtrate by PEG_6000_ precipitation, and finally, banding twice on CsCl gradients. The plaque assay method followed that was described previously for phiH [23].

### 2.2. DNA Sequencing and Assembly of the ChaoS9 Genome

The ChaoS9 genome was sequenced by the whole-genome shotgun approach (7-fold coverage). Briefly, DNA was randomly sheared by sonication and fragments cloned into plasmid vectors and sequenced by the chain termination method using the BigDye system (Applied Biosystems, ABI, Foster City, CA, USA). Contig assembly used the Phred–Phrap–Consed package [25]. The remaining gaps were sequenced by targeted PCR amplification of viral DNA using custom primers (Supplementary Table S1), followed by sequencing of the amplimers using the BigDye system. Sequencing was performed at the MPI of Biochemistry core sequencing facility (Martinsried, Germany).

### 2.3. DNA Resequencing of Natrialba Virus phiCh1

The genome sequence of phiCh1 was determined using the Illumina HiSeq platform (Max-Planck Genome Centre, Cologne, Germany), as previously described for phiH1 [26]. This generated 309 Mbp of high-quality sequence data. Reads were mapped to the reference genome (GenBank:AF440695) using the “map to reference” option within the Geneious (version 10.2) environment, as described for phiH1 [26]. Average genome coverage was 3192-fold. Gene annotation used a combination of gene prediction with GeneMarkS-2 [27] and manual refinement using database searches (BLASTp/BLASTn). Refinement of the annotation took into consideration the original annotation of phiCh1, the annotation of *Nab. magadii* plasmid pNMAG03 [28] and the annotation of haloviruses phiH1 and ChaoS9, as well as the nr database at the NCBI webserver (https://blast.ncbi.nlm.nih.gov, accessed 10 December 2018).

### 2.4. Electron Microscopy of Virus

Samples of purified ChaoS9 virus were fixed iso-osmotically with 2.5% (*v*/*v*) glutaraldehyde. A drop of the sample was then placed on a carbon-coated copper grid, freshly treated by glow discharge to make it hydrophilic. After incubation for 2 min, the drop was quickly removed, and the grid was stained with a solution of 1% (*w*/*v*) uranyl acetate and 0.01% (*w*/*v*) glucose. Micrographs were taken with an EM 912 electron microscope (Zeiss, Oberkochen, Germany) equipped with an integrated OMEGA energy filter operated in the zero-loss mode. Head diameters were measured on micrographs from vertex-to-vertex, not including the axis in line with the tail.

### 2.5. Protein Analyses of Purified Virus

Samples of purified virus were added to Laemmli sample buffer (with 2-mercaptoethanol) [29] and heated at 95 °C for 5 min before loading on a precast NuPAGE 4%–12% Bis-Tris polyacrylamide gel (Invitrogen). The running buffer was NuPAGE MES buffer with 0.1% (*w*/*v*) sodium dodecyl sulfate (SDS). PageRulerTM prestained protein ladder—3 (Fermentas, #SM1819), containing proteins of 250, 130, 100, 70, 55, 35, 25, 15, 10 kDa, were loaded in parallel. The gel was stained with Coomassie blue and destained in 10% acetic acid. 

### 2.6. Bioinformatics Analyses

Sequence alignments, editing, and phylogenetic tree reconstructions were performed within the Geneious (version 10.2) suite of programs (https://www.geneious.com/) [30]. For phylogenetic tree reconstructions, protein sequences were first aligned using CLUSTALW, and trees inferred using the Neighbor–Joining algorithm (within Geneious). Consensus trees were determined after 100 bootstrap repetitions. GeneMarkS-2 [27] was used for gene prediction. Protein and DNA sequence similarity searches used the programs BLASTp and BLASTn to search the nr databases at the NCBI webserver (https://blast.ncbi.nlm.nih.gov, accessed on 10 December 2018). The VIRFAM webserver (http://biodev.cea.fr/virfam/) [31] was used to classify ChaoS9. Halovirus genomes were compared by the dot plot method zPicture [32]. Average Nucleotide Identity (ANIb) values between viral genomes were calculated using EZbiocloud webserver [33], and a heatmap produced from these values using heatmapper [34]. Searches for matching CRISPR spacers were performed at the CRISPRs web server (http://crispr.i2bc.paris-saclay.fr/crispr/BLAST/CRISPRsBlast.php) [35] and at the IMG/VR server (https://img.jgi.doe.gov/cgi-bin/vr/main.cgi) [36], and also by direct searching of hypersaline metagenomes using the crass software [37], as previously described [26]. Identification of the *pac* site utilized the program PhageTerm [38] as implemented on the CPT Phage Galaxy (https://cpt.tamu.edu/galaxy-pub/). Correction of the molecular weight estimates of acidic proteins was based on the study of Guan et al. [39]. The following equations were used to convert the protein MW calculated from the inferred protein sequence into an apparent protein MW expected upon SDS-PAGE: MW_real_ + Sh = MW_app_, where MW_real_ is the MW computed from the protein sequence, MW_app_ is the MW expected to be observed by SDS-PAGE, and Sh is the shift (or estimated error) computed by the formula Sh = len × (276.5*x* − 31.33). Here, len is the length of the protein (in aa) and *x* represents the proportion of acidic amino acids (Glu, Asp).

#### Data Availability

The ChaoS9 genome sequence has been deposited at Genbank under the accession MK310226. The revised phiCh1 genome sequence has been deposited under accession MK450543 and the phiCh1 raw reads were submitted to the SRA and can be retrieved via BioProject PRJNA517034.

## 3. Results

### 3.1. Isolation of Halovirus ChaoS9

**Figure 1:**
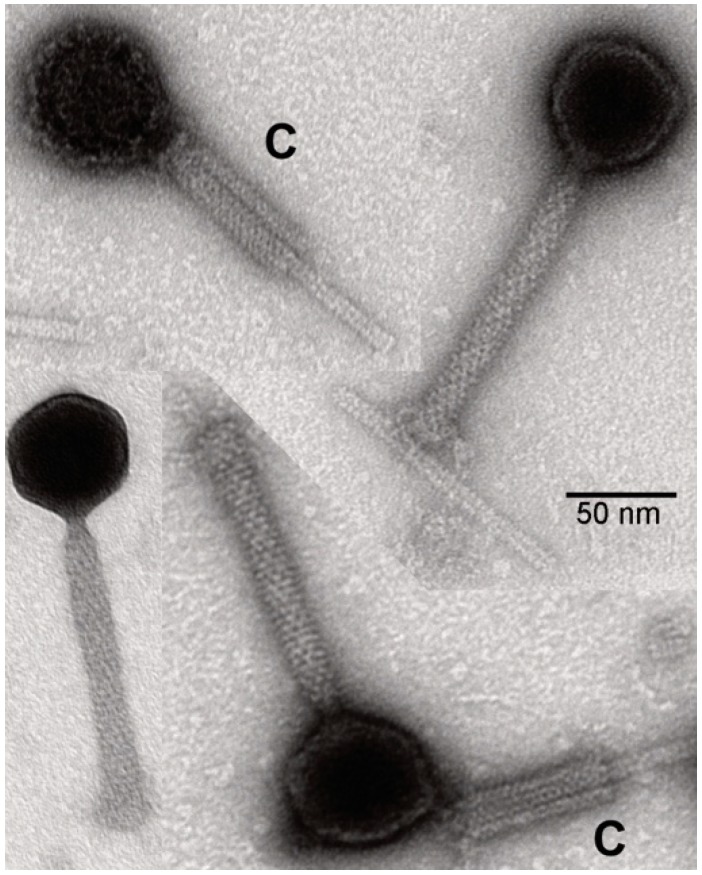
Morphology of ChaoS9 particles by negative-stain electron microscopy. Purified virus was fixed with 2.5% glutaraldehyde, negatively-stained with 1% uranyl acetate and examined under a Zeiss EM 912 electron microscope. Scale bar represents 50 nm. Examples of contracted tails (labeled with a C) are shown in the top left-hand corner and the bottom right-hand corner.

In 2007, a large-scale culture (1 m^3^) of *Hbt. salinarum* S9 lysed spontaneously. Suspecting a virus infection, the lysate was processed by the method described for purifying halovirus phiH1 [23]. This involved filtration through diatomaceous earth, addition of PEG_6000_ to precipitate virus particles, and the resulting pellets applied to CsCl gradients. Virus bands were observed on CsCl gradients, and negative-stain electron-microscopy of this fraction revealed a homogeneous population of tailed virus particles, some displaying contracted tails (Figure 1). The head diameter was 61 nm, uncontracted tails were 128 × 17 nm, and contracted tails had sheaths of 74 × 23 nm.

### 3.2. Virus Proteins

**Figure 2:**
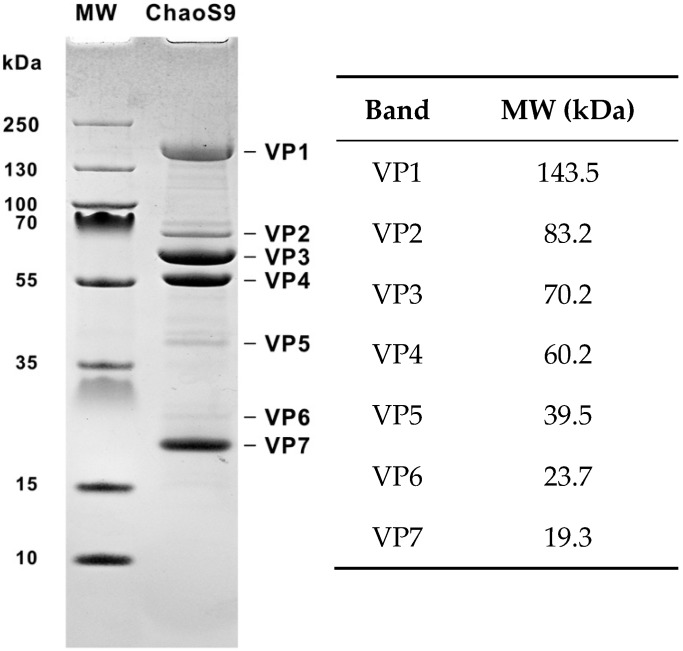
Proteins of halovirus ChaoS9. Purified virus was heated in sample buffer and separated on a 4%–12% SDS-polyacrylamide gel, and the separated proteins stained with Coomassie blue. MW, molecular weight standards (PageRuler). The sizes of the protein standards are indicated at the left (in kDa). Virus proteins (VP1 to VP7) are numbered from largest to smallest. Molecular weight estimates of the major virus proteins are shown in the adjacent table.

The proteins of purified virus were separated by SDS-PAGE and revealed four major protein bands (VP1, VP3, VP4, and VP7), three minor bands (VP2, VP5, and VP6), and several very faint bands (Figure 2). 

### 3.3. ChaoS9 Genome and Sequence

**Figure 3:**
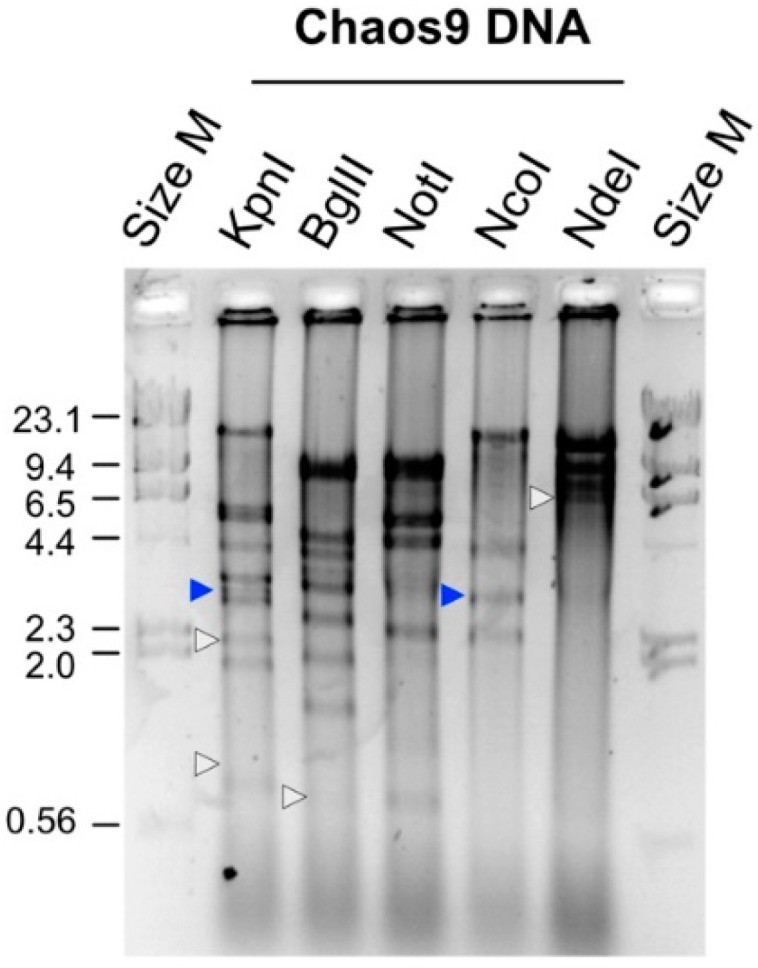
Restriction digests of ChaoS9 DNA. Enzymes used are indicated above each well. The outside wells (Size M) were loaded with DNA size markers (Lambda-HindIII), and the fragment sizes (in kbp) are shown at the left edge. White triangles; terminal fragments predicted from the DNA sequence that were either underrepresented or not visible on gels. Blue triangles; bands predicted from the DNA sequence to occur only in longer than unit length genomes. See also Supplementary Figure S1.

Nucleic acids were extracted from virus preparations, treated with several restriction enzymes and the digests separated by agarose gel electrophoresis (Figure 3). Cleavage of the viral genome by these enzymes, and the different fragment patterns observed for each enzyme, indicated that the genome was dsDNA. The ChaoS9 genome was sequenced using the whole-genome shotgun approach (7-fold coverage; see Methods) and contig gaps were closed by PCR amplification of viral DNA using specific primers (Table S1). All sequences assembled to a single contig with a unit length of 55,145 nt and a G + C content of 65.3% (Table 1). Comparison of the observed restriction fragment patterns with in silico predictions based on linear and circular versions of the genome (Figure S1) not only showed a close correspondence, but also identified terminal fragments that were either underrepresented or not visible (white triangles) on gels, as well as bands predicted to occur only in longer than unit length genomes (blue triangles). These results were consistent with the viral genome being partially redundant and circularly permuted, as is typical for headful packaging.

**Figure 4:**
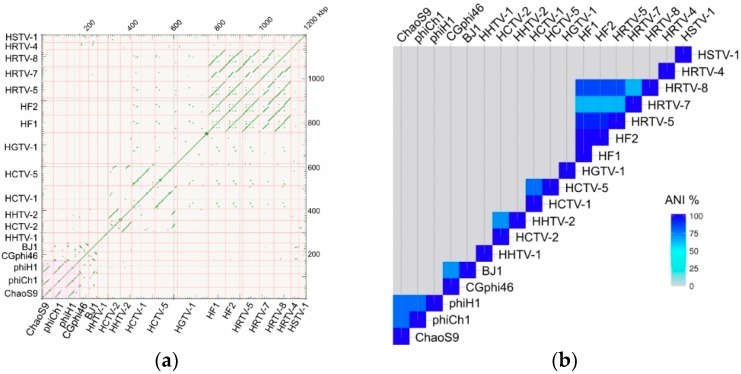
Comparison of the ChaoS9 genome with the genomes of 17 other published halovirus isolates. (**a**) Dot plot showing sequence similarity using the method of zPicture (Blastz) [32]. Names of viruses are shown along lower and left edges, and their lengths are indicated by the pink dashed lines. The scale, in kbp, is shown along the upper and right edges. The pink shaded box at lower left highlights the similarity between ChaoS9, phiCh1 and phiH1. (**b**) Average Nucleotide Identity (ANIb) heatmap. ANIb percentage values were calculated using EZbiocloud [33] and the heatmap produced using heatmapper [34]. Color scale indicates % ANIb.

**Figure 5:**
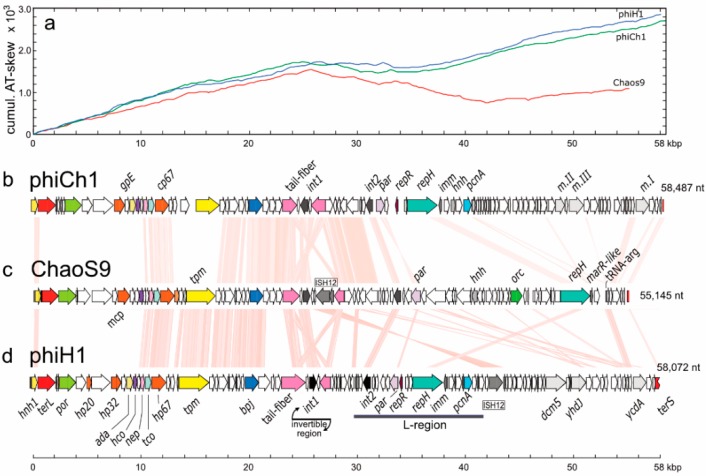
Genome map of ChaoS9 compared to phiCh1 and phiH1. (**a**) Cumulative AT-skew of all three viral genomes. (**b**) Genome map of phiCh1; (**c**) Genome map of ChaoS9; (**d**) Genome map of phiH1. Scale bar at bottom shows length, in kbp. Pink shading between genome maps indicates regions of similarity (tBLASTx, ≥30% amino acid identity). Functionally similar genes have been colored the same and are labeled nearby on one or more of the genomes (see Table 2 for details). The colors, gene labels (and encoded proteins) are: red, *terL* (large subunit terminase); light green, *por* (portal protein); brown, capsid protein genes, such as *mcp*, *gpE* and *hp32* (major capsid protein), hp20, hp67, cp67; yellow, *tpm* (tape measure protein); blue, *bpj* (baseplate J family protein); pink, tail fiber genes; light purple, *par* (plasmid partition); dark green, *orc1*, (replication protein Orc1); light blue, *repH* (plasmid replication protein); light grey, *dcm5*, *yhdJ*, *ycdA*, *m.II*, *m.III*, *m.I* (methyltransferases); dark grey, ISH12 transposase; black, integrases (*int1*, *int2*). Genes of unknown or uncertain function are uncolored (white). The L-region of phiH1 has been described by [41].

A dotplot comparison of this sequence with 17 other tailed haloviruses showed a specific and close relationship with phiCh1 and phiH1 (Figure 4a), and average nucleotide identity (ANIb) values between ChaoS9, phiCh1 and phiH1 were ≥74% (Figure 4b). In a recent study, the complete genome sequence of phiH1 was compared to the previously published sequence of phiCh1 [26], and they shared 63% (BLASTn) nucleotide identity. As part of the present study, we have resequenced phiCh1 (see below and Table S2 for details). Since phiH1 is a valid species of the genus *Myohalovirus* [40], phiCh1 should be placed in the same genus. While phiCh1 and phiH1 show strong similarity over most of their genomes, their similarity to ChaoS9 is largely confined to a central region covering from about 10–30 kbp. This can be seen in Figure 4a, but is more clearly evident in the annotated genome comparison depicted in Figure 5. 

The *pac* site of the ChaoS9 genome was identified by alignment with phiH1 and phiCh1, for which *pac* sites have been previously reported [26]. Like the other two haloviruses, *pac* occurs within the *terS* gene, near the stop codon, at a well conserved GC-rich sequence motif. For convenience, base 1 of ChaoS9 was chosen so that it corresponds with the starting bases of phiH1 and phiCh1, which places the *pac* site terminal base at nt 46. A summary of the major features of ChaoS9 is given in Table 1, along with the characteristics of phiH1 and phiCh1.

Annotation of the ChaoS9 genome predicted 85 coding sequences (CDS) and one tRNA gene (Figure 5 and Table 2). Most CDS were closely spaced, with 31 overlapping at stop/start codons, and 30 separated by 0–15 nt. The majority of CDS were organized into groups having the same orientation, such as 0–25 kbp and 42–55 kbp, where all but one CDS are on the upper (forward) strand, and 33.5 to 41.9 kbp where all CDS are on the lower (reverse) strand. The most common stop codon was TGA (56; 65%), followed by TAA (19; 22%) and TAG (10; 12%), a pattern that is similar to the host species, *Hbt. salinarum*, that also prefers TGA stop codons (TGA, 49%; TAG, 28%; TAA, 23%) (see http://www.kazusa.or.jp/codon).

The ChaoS9 open reading frames (ORFs) were compared (BLASTp) to those from phiH1 and phiCh1 to identify homologs (E-values ≤ 10^−10^). In some cases, this threshold was relaxed because there was additional support from a conserved gene neighborhood; in other cases, a more stringent threshold was applied in case of casual matches caused by a strong compositional bias. The same process was used to identify homologs in haloarchaeal proviruses (see Section 3.6).

### 3.4. Resequencing the Genome of Halovirus PhiCh1

Given the close similarity of phiCh1 to ChaoS9, it was decided to check the phiCh1 genome sequence by high throughput sequencing using Illumina HiSeq (see methods). This revealed a number of differences to the existing sequence (Genbank: AF440695.1), which are listed in Table S2 along with the genes and proteins affected. Briefly, a total of 40 bases were affected by the revision; 13 point mutations, 9 one-base indels, and one 18 base indel. Overall, the sequence revision made phiCh1 more similar to phiH1 and to ChaoS9. The *pac* terminal base was determined to be nt 46 (*p* = 1.23 × 10^−23^), based on analysis of the Illumina reads using the program PhageTerm [38]. This position is consistent with previous studies [26,43].

### 3.5. Organisation of the ChaoS9 Genome

A gene map of ChaoS9 (Figure 5, panel c) is shown between the maps of phiCh1 and phiH1 (panels b and d). Pink shading between the maps indicates regions encoding similar proteins (≥30% aa identity). Over the first 25 kbp, the three viruses share a similar gene synteny, while beyond 25 kbp, ChaoS9 differs considerably from the other two viruses in both gene composition and order. The same pattern is reflected by cumulative AT-skew plots (panel a), which show a similar, steady rise over the first 25 kbp for all three virus genomes, but after this, the plot for ChaoS9 diverges significantly from those of phiCh1 and phiH1. In general, the cumulative AT-skew plots appear to parallel the transcription directions of genes of the three viruses. 

*The left arm (0–28 kbp) of the ChaoS9 genome*. All genes are in the forward direction (top strand) and form a long, functional module specifying proteins putatively involved in DNA packaging, virus structure, and assembly. They include genes encoding the large subunit terminase (TerL) and portal protein (Por), the major virus capsid protein (Mcp), tail sheath, tail-tube, and tape measure (Tmp) proteins, and the tail fiber protein. The gene for the latter protein is also part of an invertible region (see next section). In phiCh1 and phiH1, these genes are expressed during the late phase of lytic infection [44]. The major capsid protein and the tail sheath protein are likely to produce the most prominent bands on SDS-PAGE, which were VP3 and VP4 (Figure 2). The protein molecular weights of Mcp and of the tail sheath protein calculated from their amino acid sequences were 42 kDa (Mcp) and 46.1 kDa (tail sheath), but these values are considerably lower than the observed MWs of VP3 and VP4 (70.2 and 60.2 kDa, respectively). After applying the compensatory adjustment for acidic proteins reported by Guan et al. [39], the predicted gel sizes of the Mcp (50.4 kDa) and tail sheath protein (56.1 kDa) were still lower than VP3 and VP4. The MW of the tail tube protein (14.8 kDa), after applying the Guan et al. adjustment [39] was predicted to be 19.3 kDa on SDS gels, a value identical to that of VP7. The VP1 band (143.5 kDa) is much larger than any of the annotated virus structural proteins, and may represent a multimeric form.

Sequences of head-neck-tail module proteins can be used to classify caudoviruses [45], and this classified ChaoS9 within the Myoviridae (Type1, Cluster 6). While the gene composition and synteny were well conserved between ChaoS9 and the other viruses, the sequence similarity of genes and proteins revealed major differences between them, suggesting a long history of recombination. For example, the proteins encoded by genes *terL* to *hco* of ChaoS9 showed no significant similarity to the corresponding proteins from phiCh1 or phiH1, and this segment includes many of the most highly conserved genes used for virus classification, such as the terminase, portal, and major capsid proteins. The putative assignments of ChaoS9_015 as portal protein and ChaoS9_030 as prohead protease are based on VIRFAM predictions. From 10–25 kbp, the majority of the encoded proteins are related to tail assembly proteins, and most share sequence similarity with the corresponding phiH1 and phiCh1 proteins, with the obvious exceptions (see Figure 5) of the phiCh1 tape measure protein (Tpm) and two hypothetical proteins (PhiH_155, PhiH_160) of phiH1 that immediately precede the tail fiber protein (PhiH_165).

*The invertible region (23–29 kbp)*. The inflection in AT-skew at around 25 kbp occurs at the end of the tail-fiber gene, which is embedded in a segment containing an integrase/recombinase and another tail-fiber related gene (probably a pseudogene) that is inversely oriented to the first one. The similarity of the two fiber genes (pink arrows in Figure 5) can be seen by the crossing of shaded lines (light pink shaded) in this region of Figure 5. Similar gene arrangements to this are found in phiH1 and phiCh1, where it has been shown that the central recombinase allows inversion of the nearby tail-fiber genes, so altering the sequence of the actively expressed copy [46]. The ChaoS9 invertible region contains an ISH12 transposon that is not present in the corresponding invertible regions of the other viruses. This transposon is identical to ISH12 from strain R1 and has targeted the inactive copy of the tail fiber protein (N-term part: ChaoS9_195, C-term part: ChaoS9_160). Curiously, the same transposon is also integrated into phiH1, but at a different genome position.

*The right arm (29–55 kbp).* This region is the most divergent compared to phiCh1 and phiH1, and contains genes putatively involved in replication (*parA*, *orc1* and *repH*), a tRNA-Arg gene, and numerous genes specifying proteins of unknown function. In both phiH1 and phiCh1, this region has been shown to control lysogeny and the provirus state, maintaining the viral genome as a circular, extrachromasomal dsDNA element [47,48,49]. Lytic phase gene expression in phiH1 has been shown to be repressed by RepR, a coliphage-like repressor [50,51,52], but a homologous gene similar to this was not detected in ChaoS9.

There are no DNA methylase genes in ChaoS9, while the other viruses each carry three (e.g., m.I, m.II, and m.III of phiCh1). The corresponding regions of phiH1 and phiCh1 are similar to each other, but contain relatively few genes with matching protein sequences to ChaoS9, and even in these cases the arrangement usually differs. For example, the ISH12 elements of ChaoS9 and phiH1 are 15 kbp apart, in opposite orientation, and in distinct modules. Also, while the RepH proteins show weak similarity to each other, the position of *repH* in ChaoS9 is about 15 kbp further right compared to the *repH* genes of phiCh1 and phiH1. Even the type of replication related genes differs, with ChaoS9 carrying a gene similar to Orc (*orc1*) that is not present in the other viruses, while phiH1 and phiCh1 carry a gene similar to PCNA (*pcnA*) that is not found in ChaoS9. In phiH1, the L-region has been shown to be able to replicate independently as a plasmid [41], and also contains an immunity gene (*imm*) near *repH* that protects L-plasmid containing host cells from lytic infection by phiH1 [41]. The same arrangement is found in phCh1. While a gene related to *imm* was not detected near *repH* of ChaoS9, a gene specifying a MarR-like repressor (ChaoS9_390) is present just downstream of *repH*.

ChaoS9 is the only virus of the three predicted to carry a tRNA gene, tRNA-Arg(TCG). In BLASTn searches, this sequence is unlike other tRNAs except for a conserved region of the right arm (nt 43-70), which matches many haloarchaeal and bacterial tRNAs. Curiously, the best matches are to cyanobacteria tRNAs, such as tRNA Gly (CCC) of *Synechococcus* sp. KORDI-100 (CP006269 nt 191738-191667), which gives a 28 nt perfect alignment. In haloarchaea, the best match found was 19 nt. Although the ChaoS9 tRNA appears to be complete, its function is less clear. Ostensibly, it specifies arginine (anticodon TCG), and the corresponding codon is the third most frequent Arg codon used in both the host species *Hbt. salinarum* [53] and ChaoS9, but there is no large difference in usage between the two (7% in *Halobacterium* and 11% in ChaoS9), and there are much rarer codons for this amino acid used by both host and virus. It could also represent an *att* sequence used for integration of the viral genome into tRNA genes of host strains (see later) or may have a regulatory role. 

### 3.6. Related Provirus-Like Matches in Haloarchaea

**Figure 6:**
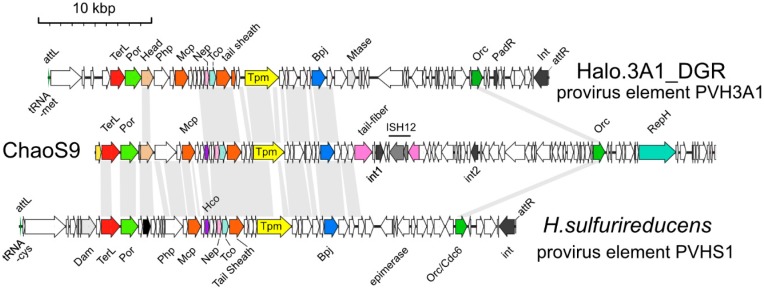
Provirus elements PVH3A1 and PVHS1 compared to ChaoS9. PVH3A1 from haloarchaeon strain 3A1_DGR (accession NZ_KK033114; nt 1475908-1520418), and PVHS1 from *Haa. sulfurireducens* strain M27-SA2 (accession NZ_CP011564; nt 1971038- 1927125), are compared to ChaoS9. Grey bands connecting genome maps represent similarity (tBLASTx, >30% identity) between the inferred proteins of ChaoS9 and each provirus. Positions of several annotated proteins and their genes are indicated by color and name; TerL, large subunit terminase (red); Por, portal protein (light green); Head, SPP1_gp7 family head assembly protein, (light brown); capsid proteins such as Mcp (major capsid protein) and Tail sheath protein (brown); Tpm, tape measure protein (yellow); Dam, adenine methyltransferase (light grey); Bpj, baseplate J family protein (blue); Orc1, replication protein Orc1 (green); RepH, plasmid replication protein (turquoise). Scale bar, in kbp, is shown at top.

Several haloarchaeal genomes carried provirus elements related to ChaoS9, and the gene maps of two examples (PVH3A1 and PVHS1) are depicted in Figure 6. The *attL* and *attR* sites of both proviruses indicate they were integrated into tRNA genes (tRNA-Met and tRNA-Cys), probably mediated by the integrases encoded at their right ends. Both appear to be intact and probably functional, as their left halves possess complete suites of virus structural and assembly genes. Their right halves predominantly carry genes for uncharacterized proteins unrelated to ChaoS9. A few genes in this half, and at the extreme left end (before the structural/assembly genes), could be assigned functions, such as the replication protein Orc, and DNA methylases (Mtase, Dam). Neither provirus carried a region corresponding to the invertible tail-fiber genes of ChaoS9.

The right arm (29–55 kbp) of ChaoS9 shows little similarity to either provirus except for Orc1. Across the left arm (0–28 kbp), the region covering virus structural and assembly proteins, ChaoS9 maintains good synteny and protein similarity to PVHS1, but with PVH3A1 there is a distinct break in similarity within the virus structural/assembly gene module. PVH3A1 neck and tail proteins are similar to ChaoS9 but the head/assembly proteins are unrelated, except for the head assembly protein (labeled Head; Figure 6, panel a). PhiH1, and to a lesser extent phiCh1, show a comparable break in similarity to ChaoS9 head and tail genes (Figure 5). All of these examples suggest that ancestral recombination events between ChaoS9-like viruses have occurred within genes located between the head morphogenesis genes and the tail morphogenesis genes, and that these often result in viable progeny.

The distinct right halves of the proviruses suggest that they have also undergone extensive recombination relative to ChaoS9, perhaps reflecting differences in virulence and/or provirus state. 

### 3.7. A Diverse Family of Haloviruses

**Figure 7:**
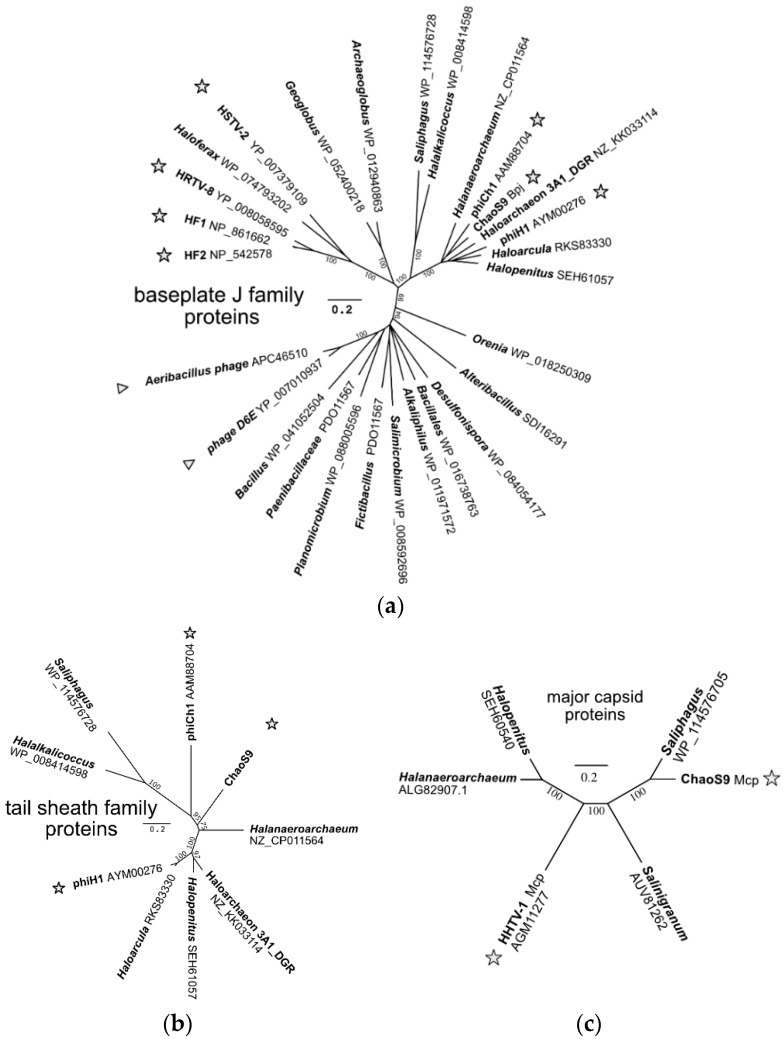
Phylogenetic tree reconstructions using (**a**) baseplate J family proteins, (**b**) major tail sheath proteins and (**c**), major capsid proteins. Halovirus proteins are marked by stars and bacteriophage proteins are marked by triangles. The consensus trees were produced using the Neighbor–Joining algorithm and 100 bootstrap replications. Scale bars represent the estimated number of amino acid replacements per position.

In order to examine evolutionary relationships between ChaoS9 and other viruses and proviruses, genes were sought that were both sufficiently conserved and present in all examples. As shown in Figure 5 and Figure 6, relatively few genes matched these criteria. Even the large subunit terminase (TerL) and major capsid protein (Mcp) were poorly conserved, and these have been widely used in previous studies of bacteriophages for delineating virus taxa [4]. The Bpj (baseplate J family protein) and the tail sheath proteins were selected to infer phylogenetic relationships as they were relatively long, conserved in sequence and present in all examples. BLASTp searches (accessed 20 November 2018) with ChaoS9 Bpj retrieved over 50 high scoring matches (E value < 10^−30^), with the closest matches all being from haloarchaea or haloviruses, while less similar matches included proteins from Bacteria and bacteriophages. An inferred phylogenetic tree based on alignment of Bpj (Figure 7a) shows that ChaoS9 is part of a robust clade (100% bootstrap confidence) which includes haloviruses phiCh1, phiH1, and provirus-like elements of four haloarchaea belonging to at least three different genera. A separate clade, also with high bootstrap confidence, contains four haloviruses (HF1, HF2, HRTV-8, and HSTV-2) and a provirus element of *Haloferax larsenii*.

BLASTp searches of the NCBI database with the ChaoS9 tail sheath protein (ChaoS9_080) retrieved only nine high scoring matches (E value < 10^−35^), and these were all from haloarchaea or haloviruses present in the Bpj tree, and most closely related to ChaoS9. The inferred phylogenetic tree based on this protein (Figure 7b) reveals a topology similar to that of Bpj proteins. 

The major capsid protein (Mcp) of ChaoS9 was used to search the NCBI database (BLASTp, nr database, accessed 22 November 2018) and retrieved only five matches (Figure 7c), which varied in similarity from 36 to 76% (aa identity). Three were from organisms previously identified as specifying ChaoS9-related proteins (*Haa. sulfurireducens*, *Saliphagus* sp. LR7 and *Hpt. malekzadehii*) and are present in Figure 6 and Figure 7, while the other two sequences were from *Salinigranum rubrum* and the tailed halovirus HHTV-1 [5,54]. 

The DNA sequences of HHTV-1 and ChaoS9 share no significant similarity (Figure 4), but a BLASTp comparison of all ChaoS9 and HHTV-1 proteins found that Mcp was the only protein with significant similarity (36%) shared between these haloviruses. Such a pattern of similarity between tailed viruses that is strictly limited to the Mcp appears to be uncommon in the published literature. A less clear-cut but comparable example occurs between actinophages Jace and Tweety (accessions EF536069 and MH153804), which share similar Mcp (32%) and integrase (39%) protein sequences but weak or negligible similarity between all other proteins. Cases where head genes and tail genes derive from different virus lineages are slightly more common [55,56] (see Discussion). 

### 3.8. CRISPR Spacer Matches to ChaoS9

The ChaoS9 genome was used to search for matching CRISPR spacer sequences available from public databases (see methods). A total of 277 spacers were identified that closely matched ChaoS9, with the majority of spacers originating from Antarctic lake metagenomes (Deep Lake, Rauer Lake and Club Lake). After removing duplicates, the number of distinct spacers was reduced to 39, and in Table S3 the matching spacers have been ordered by their position along the ChaoS9 genome. The distribution of spacers is highly skewed, with most (34/39) targeting sequences within the right arm, and particularly the *repH* gene, for which there were 14 distinct spacer sequences. The direct repeats (DR) of the matching spacers were most similar or identical to those found in CRISPR arrays of sequenced haloarchaea, particularly antarctic isolates such as *Halorubrum lacusprofundi*, *Halobacterium* sp. DL1 and haloarchaeon DL3. For comparison, the phiH1 and phiCh1 genomes were also scanned for matching spacers in the same antarctic lake metagenomes (IMG/VR webserver; (https://img.jgi.doe.gov/cgi-bin/vr/main.cgi; accessed 20 December 2018). This returned only 2 (phiCh1) and 3 (phiH1) significant matches (supplementary Table S4), of which all of the phiH1 matching spacers were to the tail fiber gene (PhiH1_165), as was one of the spacers matching phiCh1 (PhiCh1_155). The remaining spacer to phiCh1 matched a sequence within the prohead protease gene (gpB, PhiCh1_045). All six spacers were from Deep Lake and Rauer Lake metagenomes. 

## 4. Discussion

This study focused on identifying the cause of a lysis event in a large, laboratory culture of *Hbt. salinarum* S9. A novel myovirus was recovered, ChaoS9, with morphological and molecular characteristics specifically resembling myoviruses phiH and phiCh1, but which differed significantly in sequence from both of these. An earlier lysis event in the same laboratory that affected a culture of *Hbt. salinarum* R1 was shown to have been caused by phiH, however, the 2007 event was not a recurrent infection. The sources of both infections are unknown, possibly raw salt, but these events highlight the need for preventative measures, even though the high salt conditions used for cultivation of haloarchaea are generally regarded as providing a strong barrier to contamination by non-halophilic microorganisms. However, tailed viruses (caudoviruses) such as ChaoS9 are not only the most common type of prokaryotic virus, but together with other bacterial and archaeal viruses, they represent the most abundant biological entity on Earth, estimated to be 10^31^ virions [57]. It is not surprising, then, that virus contamination and lysis events are a constant threat in large-scale commercial fermentations [22], and cause such significant losses that systematic preventative programs have been formulated, such as PACCP (phage analysis and critical control point) [58]. 

ChaoS9 was most similar in morphology, genome type, genome length, and sequence to the tailed haloviruses phiH1, and phiCh1 [26,43]. The dsDNA genome was terminally redundant and circularly permuted, consistent with headful packaging, as has been shown for the related viruses. The likely *pac* site was identified by sequence homology. Gene synteny of the virus morphogenesis genes of the left arm of the genome was similar to those of phiH1 and phiCh1, while the right arm comprised genes for DNA replication, plasmid partition, and a tRNA, as well as many genes specifying proteins of unknown function. The right arm corresponds to the lysogeny, replication, and accessory gene region of phiH1 and phiCh1, and probably serves the same general function in ChaoS9. A characteristic feature of phiH1 and phiCh1 is an invertible tail fiber gene module, which was also present in ChaoS9, except that it also included an ISH12 transposon. 

Comparison of the ChaoS9 genome with those of phiH1 and phiCh1 revealed a distinctive pattern of similarity and difference, suggesting an evolutionary history involving large recombination events. While the tail gene region of ChaoS9 was similar to the other two viruses, the head/assembly genes of the left arm of the genome, as well as most of right arm (the replication/lysogeny region), were not. This means that the major capsid protein, terminase (large subunit) and portal proteins of ChaoS9 are all unrelated to those of phiH1 and phiCh1. The most parsimonious explanation is that a recombination event has replaced the head morphogenesis module while leaving the tail morphogenesis module intact. It appears to be a chimeric recombinant, with the point of similarity disjuncture occurring between the putative head closure (*hco*, ChaoS9_055) and neck protein (*nep*, ChaoS9_070) genes. Similar examples where head morphogenesis and tail morphogenesis modules appear to derive from different virus types are not uncommon in the literature, such as the *Gordonia* spp. phage Kita (Figure 4 of [55]), and *Xylella fastidiosa* phage Xfas53 [56], phi80 and Gifsy-2 [59]. The likelihood of such recombination events producing viable progeny is increased because of the way caudoviruses are assembled, with heads and tails produced as separate structures that are then joined together [60]. 

Provirus elements related to ChaoS9 were found among haloarchaeal genome sequences, integrated into their host chromosomes via tRNA genes, which supports the view that ChaoS9, like phiH1 and phiCh1, has a temperate lifestyle [61]. One provirus (PVHS1) showed good sequence similarity to ChaoS9 proteins across most of the virus morphogenesis genes, while a second provirus (PVH3A1) showed a general pattern of sequence similarity to ChaoS9 like that of phiH1 and phiCh1; with mainly unrelated head morphogenesis genes (including dissimilar major capsid proteins), similar tail morphogenesis genes, and largely unrelated right arms (lysogeny/replication). The right arms of both proviruses differed significantly from the right arm of the ChaoS9 genome, but all carried a putative *orc1* gene, presumably for use in replication as a (provirus) plasmid if the circularized genome is unable to integrate into the host chromosome via *attP*. These two examples again point to frequent recombination events that exchange the head morphogenesis module while retaining the tail morphogenesis genes. 

Curiously, the ChaoS9 major capsid protein is most similar to that of the haloarchaeal siphovirus HHTV-1, but this is the only protein between them that is similar, and they are otherwise unrelated. In studies of enterobacterial viruses, major capsid protein (MCP) gene exchange was estimated to be extremely rare between phage clusters or types [61]. How this occurred in the evolutionary history of these viruses will be intriguing to resolve, but presumably indicates widespread recombination between tailed viruses of haloarchaea.

A surprisingly large number of identical or near identical CRISPR spacers to ChaoS9 were detected, and even more surprising was their identification in the metagenomes from Antarctic salt lakes. The majority of target sites were located in the right arm of the genome, in the lysogeny/replication region. A few were to sites within the left arm of the genome, such as the tail fiber gene. In contrast to ChaoS9, the genomes of phiH1 and phiCh1 matched only a few spacers from the same Antarctic metagenomes, and all but one targeted the tail fiber gene. This suggests that these lakes harbor tailed viruses with a lysogeny/replication module, similar to ChaoS9. Their tail fiber genes also show significant sequence similarity to ChaoS9, phiH1, and phiCh1, but the other virus morphogenesis genes are distinct.

The precise relationship between ChaoS9, phiCh1, and phiH1 was explored in several ways. They share average nucleotide identity (ANIb) values of ≥74%, and phylogenetic tree reconstructions using baseplate J virus protein sequences clustered ChaoS9, phiH1, and phiCh1 into a well-supported clade, distinct from other tailed haloviruses. The close relationship is consistent with the other comparative data (morphology, genome size and packaging, gene synteny, and inferred protein sequences). *Halobacterium virus phiH* is the type species of the genus *Myohalovirus* and a previous study has shown that phiCh1 should be included in the same genus, as their genomes share 63% nucleotide identity, are mostly colinear, and their proteins show, on average, 70% amino acid identity [26]. PhiCh1 proteins affected by genome revision became more similar to those of phiH1. ChaoS9 shows many similarities and correspondences to phiH1 and phiCh1 but also considerable differences, including a distinct major capsid protein, terminase (large subunit) and portal protein, and is certainly a distinct species to phiH1 and phiCh1, but whether it should be classified outside of the *Myohalovirus* genus requires further consideration. 

## Figures and Tables

**Table 1:** Characteristics of ChaoS9 and related haloviruses phiH1 and phiCh1.

Virus ^1^	Head Diameter (nm)	Tail Length × Width (nm)	Morphotype	Plaque Morphology	Unit Genome Length ^2^ (nt)	%G + C	GenomeEnds in Virus ^3^	Temperate (Genome Form)
ChaoS9	61	128 × 17	myovirus	turbid	55,145	65.3	ds, linear, TR, CP, >1 unit length	?
phiH1	64	170 × 18	myovirus	turbid	58,072	63.7	ds, linear, TR, CP, >1 unit length	Yes, provirus is a plasmid (circular, ds, 1 unit length)
phiCh1	70	130 × 20	myovirus	turbid	58,487	61.9	ds, linear, TR, CP, >1 unit length	Yes, provirus is a plasmid (circular, ds, 1 unit length)

^1^ Data from this study for ChaoS9, [23] for phiH and [42] for phiCh1. ^2^ Data from this study (ChaoS9), Dyall-Smith et al. [26] (phiH1), and Witte et al., and this study [43] (phiCh1). ^3^ TR, CP; terminally redundant, circularly permuted.

**Table 2:** Annotated coding sequences (CDS) of the ChaoS9 genome (accession MK450543).

Start (nt)	Stop (nt)	Locus_Tag	Length (bp)	Direction	Gene	Product	Homologs ^1^: phiCh1, pNMAG03	Homologs ^2^: phiH1, [Other]
100	669	ChaoS9_005	570	+	-	HTH domain protein	PhiCh1_005, PhiCh1p02, ORF1, Nmag_4251	PhiH1_005
656	2302	ChaoS9_010	1647	+	*terL*	terminase large subunit TerL	-	[HALG_00007]
2316	3944	ChaoS9_015	1629	+	*por*	portal protein Por	-	[HGTV1_7]
3937	4083	ChaoS9_020	147	+	-	CxxC motif protein	-	ORPHAN
4086	5273	ChaoS9_025	1188	+	-	putative phage head assembly protein, SPP1_gp7 family	-	[C478_10461]
5384	7300	ChaoS9_030	1917	+	-	probable prohead protease protein	-	[HLASA_2034]
7303	7755	ChaoS9_035	453	+	-	uncharacterized protein	-	[HLASA_2033]
7801	8928	ChaoS9_040	1128	+	-	major capsid protein MCP	-	^3^ [HLASA_2032; HHTV1_21]
8944	9363	ChaoS9_045	420	+	-	uncharacterized protein	PhiCh1_055, PhiCh1p13, ORF12, Nmag_4261	^6^ PhiH1_050, [HLASA_2031]
9398	9784	ChaoS9_050	387	+	-	uncharacterized protein	-	[HLASA_2030]
9781	10191	ChaoS9_055	411	+	*hco*	head closure protein Hco, type 1	PhiCh1_065, PhiCh1p15, ORF14, Nmag_4263	^5^ PhiH1_060, [WP_054519912]
10188	10421	ChaoS9_060	234	+	-	uncharacterized protein	-	ORPHAN
10414	10701	ChaoS9_065	288	+	-	uncharacterized protein	PhiCh1_070, PhiCh1p16, ORF15, Nmag_4264	^6^ PhiH1_065, [HLASA_2029]
10703	11149	ChaoS9_070	447	+	*nep*	putative neck protein Nep, type 1	PhiCh1_075, PhiCh1p17, ORF16, Nmag_4265	PhiH1_070 [HLASA_2028]
11156	11746	ChaoS9_075	591	+	*tco*	tail completion protein Tco, type 1	PhiCh1_080, PhiCh1p18, ORF17, Nmag_4266	PhiH1_075 [HLASA_2027]
11767	13071	ChaoS9_080	1305	+	-	tail sheath protein	PhiCh1_085, PhiCh1p19, ORF18, Nmag_4267	PhiH1_080 [HLASA_2026]
13082	13480	ChaoS9_085	399	+	-	predicted tail tube protein	PhiCh1_090, PhiCh1p20, ORF19, Nmag_4268	PhiH1_085 [WP_054519907]
13492	13932	ChaoS9_090	441	+	-	uncharacterized protein	PhiCh1_095, PhiCh1p21, ORF20, Nmag_4269	PhiH1_090 [HLASA_2025]
13935	14144	ChaoS9_095	210	+	-	uncharacterized protein	-	[WP_054519905]
14147	16891	ChaoS9_100	2745	+	*tpm*	tape-measure tail protein Tpm	^7^ PhiCh1_105, PhiCh1p23+PhiCh1p24, ORF22+ORF23, Nmag_4272	PhiH1_100 [HLASA_2024]
16895	17422	ChaoS9_105	528	+	-	uncharacterized protein	PhiCh1_110, PhiCh1p25, ORF24, Nmag_4273	PhiH1_105 [HLASA_2023]
17423	17767	ChaoS9_110	345	+	-	uncharacterized protein	PhiCh1_115, PhiCh1p26, ORF25, Nmag_4274	PhiH1_110 [HLASA_2022]
17771	18604	ChaoS9_115	834	+	-	uncharacterized protein	^7^ PhiCh1_120, PhiCh1p27+PhiCh1p28, ORF26+ORF27, Nmag_4275	PhiH1_115 [HLASA_2021]
18644	18787	ChaoS9_120	144	+	-	CxxC motif protein	-	PhiH1_120
18784	19335	ChaoS9_125	552	+	-	uncharacterized protein	PhiCh1_125, PhiCh1p29, ORF28, Nmag_4276	PhiH1_125 [HLASA_2020]
19338	19700	ChaoS9_130	363	+	-	virus-related protein	-	PhiH1_135
19697	20062	ChaoS9_135	366	+	-	uncharacterized protein	PhiCh1_130, PhiCh1p30, ORF29, Nmag_4277	PhiH1_140 [HLASA_2019]
20069	21328	ChaoS9_140	1260	+	*bpj*	baseplate J family protein Bpj	PhiCh1_135, PhiCh1p31, ORF30, Nmag_4278	PhiH1_145 [HLASA_2018]
21321	21929	ChaoS9_145	609	+	-	uncharacterized protein	PhiCh1_140, PhiCh1p32, ORF31, Nmag_4279	PhiH1_150 [HLASA_2017]
21933	22517	ChaoS9_150	585	+	-	uncharacterized protein	PhiCh1_145, PhiCh1p33, ORF32, Nmag_4280	- [HLASA_2016]
22514	23122	ChaoS9_155	609	+	-	uncharacterized protein	PhiCh1_150, PhiCh1p34, ORF33, Nmag_4281	- [HLASA_2015]
23115	24698	ChaoS9_160	1584	+	-	repeat-containing tail fiber protein	^8^ PhiCh1_155+ PhiCh1_175, PhiCh1p35+PhiCh1p37, ORF34+ORF36, Nmag_4282+Nmag_4286	PhiH1_165+ PhiH1_185
24702	24986	ChaoS9_165	285	+	-	uncharacterized protein	^8^ PhiCh1_160+ PhiCh1_170, Nmag_4285+Nmag_4283	PhiH1_180+ PhiH1_170
25024	25698	ChaoS9_170	675	+	*int1*	tyrosine integrase/recombinase Int1	PhiCh1_165, PhiCh1p36, ORF35, Nmag_4284	PhiH1_175
25709	25996	ChaoS9_175	288	-	-	uncharacterized protein	^8^ PhiCh1_160+ PhiCh1_170, Nmag_4285+Nmag_4283	PhiH1_180+ PhiH1_170
25999	26145	ChaoS9_180	147	-	-	repeat-containing tail fiber protein (C-term) (nonfunctional)^9^	* ^9^	* ^9^
26199	27455	ChaoS9_185	1257	-	*tnpB*	IS1341-type transposase TnpB	-	PhiH1_340
27457	27849	ChaoS9_190	393	-	*tnpA*	IS200-type transposase TnpA	-	PhiH1_335
27906	28820	ChaoS9_195	915	-	-	repeat-containing tail fiber protein (N-term) (nonfunctional)	^8^ PhiCh1_155+ PhiCh1_175, PhiCh1p35+PhiCh1p37, ORF34+ORF36, Nmag_4282+Nmag_4286	PhiH1_165+ PhiH1_185
28854	29579	ChaoS9_200	726	+	-	transmembrane domain protein	PhiCh1_180, PhiCh1p38, ORF37, Nmag_4287	-
29589	29861	ChaoS9_205	273	-	-	HTH domain protein	PhiCh1_185, PhiCh1p39, ORF38, Nmag_4288	-
29933	30241	ChaoS9_210	309	-	-	uncharacterized protein	PhiCh1_190, PhiCh1p40, ORF39, Nmag_4289	PhiH1_220
30238	30813	ChaoS9_215	576	-	-	glutamine amidotransferase domain protein, class-II	PhiCh1_195, PhiCh1p41, ORF40, Nmag_4290	-
30818	31891	ChaoS9_220	1074	-	-	uncharacterized protein	^7^ PhiCh1_200, PhiCh1p42+PhiCh1p43, ORF41+ORF42, Nmag_4291	-
32030	32266	ChaoS9_225	237	+	-	uncharacterized protein	-	ORPHAN
32339	32584	ChaoS9_230	246	+	-	uncharacterized protein	-	PhiH1_225
32581	33009	ChaoS9_235	429	+	-	VapC family toxin	-	[BRC75_08225]
33098	33448	ChaoS9_240	351	+	-	uncharacterized protein	PhiCh1_230, Nmag_4297	PhiH1_250
33457	34059	ChaoS9_245	603	-	*int2*	tyrosine integrase/recombinase Int2	PhiCh1_215, PhiCh1p46, ORF45, Nmag_4294	PhiH1_240
34241	34432	ChaoS9_250	192	-	-	uncharacterized protein	-	ORPHAN
34507	35055	ChaoS9_255	549	-	-	uncharacterized protein	-	PhiH1_255 [C466_00612]
35048	35902	ChaoS9_260	855	-	-	Plasmid partition protein ParA	PhiCh1_220, PhiCh1p47, ORF46, Nmag_4295	PhiH1_265
35976	36440	ChaoS9_265	465	-	-	transmembrane domain protein	-	PhiH1_210
36460	38241	ChaoS9_270	1782	-	-	uncharacterized protein	-	[AV929_12240]
38243	38857	ChaoS9_275	615	-	-	uncharacterized protein	-	[CRI94_04435]
38850	39080	ChaoS9_280	231	-	-	CxxC motif protein	-	[HALLA_11930]
39073	39240	ChaoS9_285	168	-	-	transmembrane domain protein	-	ORPHAN
39233	40492	ChaoS9_290	1260	-	-	uncharacterized protein	-	[DM826_07215]
40485	40673	ChaoS9_295	189	-	-	CxxC motif protein	-	ORPHAN
40663	41181	ChaoS9_300	519	-	-	uncharacterized protein	-	[DJ71_18565]
41174	41611	ChaoS9_305	438	-	-	HNH-type endonuclease/MarR family transcription regulator	-	^4^ [DJ70_12900; B4589_07635]
41613	41870	ChaoS9_310	258	-	-	HTH domain protein	-	[Natpe_3999]
41997	42602	ChaoS9_315	606	+	-	uncharacterized protein	-	[C480_10020]
42605	43051	ChaoS9_320	447	+	-	uncharacterized protein	-	[Natgr_3468]
43044	43250	ChaoS9_325	207	+	-	uncharacterized protein	-	ORPHAN
43250	43621	ChaoS9_330	372	+	-	uncharacterized protein	-	[OSG_eHP13_00215]
43621	44127	ChaoS9_335	507	+	-	uncharacterized protein	-	ORPHAN
44124	44255	ChaoS9_340	132	+	-	CxxC motif protein	-	[SAMN04488133_0114]
44342	45400	ChaoS9_345	1059	+	*orc1*	Orc1-type DNA replication protein	-	[HLASA_2006]
45482	45709	ChaoS9_350	228	-	-	uncharacterized protein	-	ORPHAN
45837	45992	ChaoS9_355	156	+	-	uncharacterized protein	-	[HALDL1_16575]
46369	46905	ChaoS9_360	537	+	-	uncharacterized protein	PhiCh1_295, PhiCh1p65, ORF64, Nmag_4216	^5^ [C472_00499]
46902	47177	ChaoS9_365	276	+	-	uncharacterized protein	-	ORPHAN
47179	47979	ChaoS9_370	801	+	-	zinc-finger domain protein	-	[DJ84_18225]
47976	48068	ChaoS9_375	93	+	-	uncharacterized protein	-	ORPHAN
48065	48397	ChaoS9_380	333	+	-	uncharacterized protein	-	ORPHAN
48394	51690	ChaoS9_385	3297	+	*repH*	plasmid replication protein RepH	PhiCh1_245, PhiCh1p55, ORF54, Nmag_4299	PhiH1_285
51683	51925	ChaoS9_390	243	+	-	MarR family transcription regulator	-	[AV929_12115]
51932	52591	ChaoS9_395	660	+	-	CxxC motif protein	-	[C443_17983]
53208	53453	ChaoS9_400	246	+	-	transmembrane domain protein	PhiCh1_440, PhiCh1p93, ORF92, Nmag_4244	PhiH1_460
53446	53769	ChaoS9_405	324	+	-	transmembrane domain protein	PhiCh1_445, PhiCh1p94, ORF93, Nmag_4245	^10^ PhiH1_465
53766	54455	ChaoS9_410	690	+	-	uncharacterized protein	-	^3^ [halTADL_2427; HGTV1_34]
54460	54705	ChaoS9_415	246	+	-	DUF217 domain protein	PhiCh1_460, PhiCh1p97, ORF96, Nmag_4248	-
54734	54922	ChaoS9_420	189	+	-	CxxC motif protein	PhiCh1_465, PhiCh1p98, ORF97, Nmag_4249	PhiH1_480
54915>	<63	ChaoS9_425	294	+	*terS*	terminase small subunit TerS	PhiCh1_470, PhiCh1p01, ORF98, Nmag_4250	PhiH1_485

^1^ PhiCh1/pNMAG03 homologs of ChaoS9 proteins. For phiCh1, three codes are given: the locus tag from the revised genome (PhiCh1_), the RefSeq PhiCh1p and the originally assigned ORF codes (ORF for open reading frame). For example, PhiCh1_005, PhiCh1p02, Orf1. Codes starting with PhiCh1_ represent the revised genome sequence and annotation (Genbank accession MK450543; this publication), ORF codes represent the original annotation of the phiCh1 genome [35] (Genbank accession AF440695.1), while codes beginning with PhiCh1p represent the RefSeq version of the annotation of the same genome sequence (GB accession NC_004084). The number shift between ORF and PhiCh1p is due to the *terS* gene, the N-terminal part being encoded at the end of the genome, and the C-terminal part at its beginning. This gene is ORF98 in the original annotation and PhiCh1p01 in the RefSeq annotation. Codes starting with Nmag_ represent the annotation of the *Nab. magadii* plasmid pNMAG03 [28] (accession CP001935.1), which is the provirus state of phiCh1. The point of ring opening in pNMAG03 was set between Nmag_4303 and Nmag_4211. The absence of ORF and PhiCh1p codes indicates missing gene calls in the original annotation of phiCh1. ^2^ phiH1 homologs, or else “other” homologs of ChaoS9 proteins. If a homolog exists in phiH1 then the code is provided; if a homolog is lacking from phiH1 but exists in phiCh1, this is indicated by a hyphen; if a homolog is lacking in both, phiH1 and phiCh1, then an existing “other” homolog is listed in square brackets; codes are either from UniProt (locus tags) or from NCBI nr (WP numbers). The term ORPHAN indicates a complete lack of homologs. ^3^ Multiple homologs, separated by semicolon, are listed when a homolog is found in a halovirus, but this is significantly more distant than the closest homolog. ^4^ Multiple homologs indicate a ChaoS9-specific gene fusion. ^5^ The combination of phiH1/phiCh1 and “other” homologs is used when the homologs from phiH1 or phiCh1 are especially distant. ^6^ The combination of phiH1/phiCh1 and “other” homologs is used for HLASA_ codes to illustrate a longer stretch of synteny to PVHS1 from *Halanaeroarchaeum sulfurireducens* (see later). ^7^ Multiple PhiCh1p/ORF codes indicate that the gene was split by a frameshift in the originally published genome sequence of that virus. ^8^ Multiple PhiCh1/Nmag codes indicate the existence of paralogs. ^9^ This ORF represents the C-terminal fragment of a pseudogene (indicated by the term nonfunctional) which has been targeted by ISH12. The asterisks (*) indicate that corresponding pseudogene fragments do not exist as independent ORFs in phiH1 or phiCh1. ^10^ PhiCh1_445 and PhiH1_465, like ChaoS9_405, have three predicted transmembrane domains and are suspected to function as a holin [43].

## Supplementary Materials

The following are available online at https://www.mdpi.com/2073-4425/10/3/194/s1, Figure S1: Experimental and in silico restriction digests of ChaoS9 DNA. Table S1; DNA primers used for PCR or sequencing viral DNA. Table S2; Revisions of phiCh1 genes after re-sequencing the genome. Table S3; CRISPR spacers matching ChaoS9; Table S4, CRISPR spacers matching phiH1 and phiCh1.
