# Supplementary material for: Halobacterium salinarum virus ChaoS9, a Novel Halovirus Related to PhiH1 and PhiCh1
Source: Genes (Basel). 2019 Mar 1;10(3):194. doi: 10.3390/genes10030194 (PMC6471424; doi:10.3390/genes10030194)
Supplement: Supplementary file 1 [file genes-10-00194-s001.pdf]

Supplementary data.

Supplementary Figure S1. Experimental and *in silico* restriction digests of ChaoS9 DNA

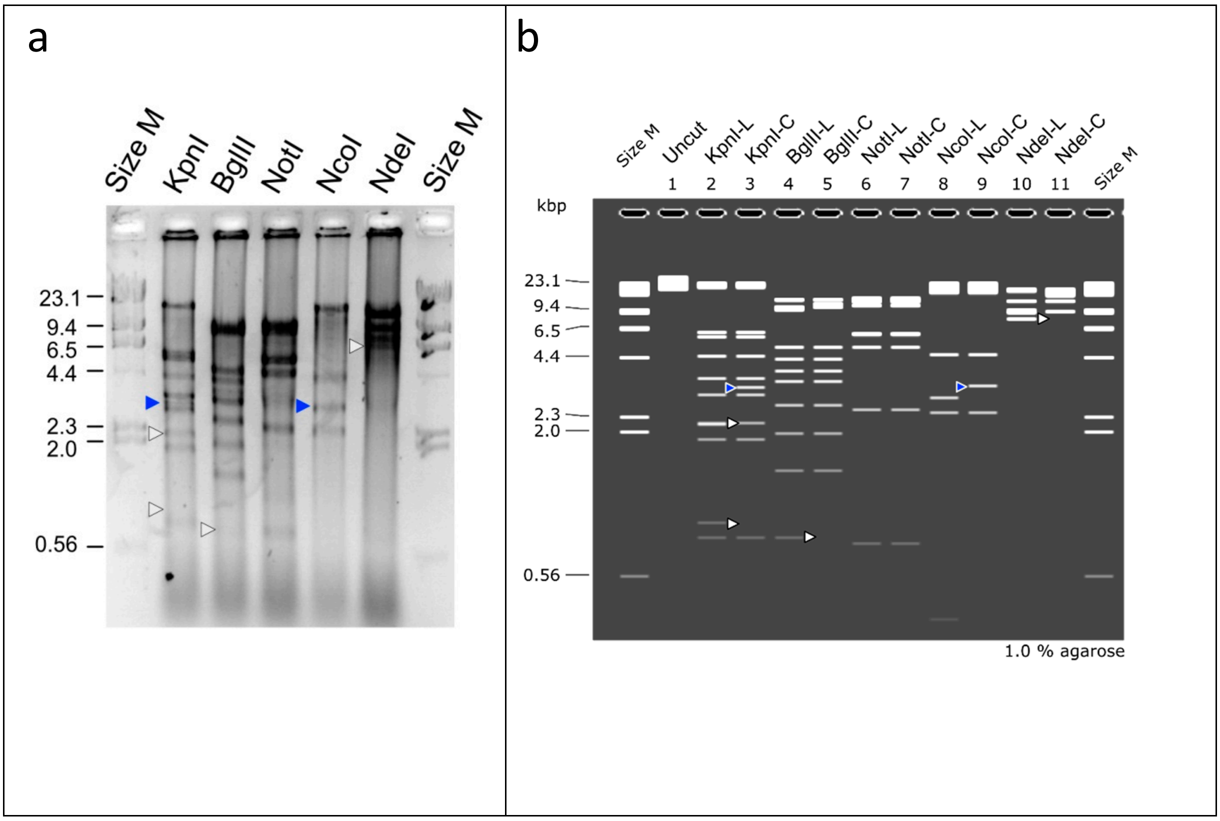

Figure S1. Agarose gel electrophoresis of restriction digests of ChaoS9 DNA (left panel) compared to a virtual gel of restriction fragments predicted for linear (L) and circular (C) forms of the genome sequence (right panel).

Left panel: Restriction digests of ChaoS9 DNA. Restriction enzymes are shown above each gel lane and size markers (HindIII-cut lambda DNA) were run in lanes marked Size M. Sizes of the marker DNA fragments are given at the left edge, in kbp. Gel was stained with ethidium bromide and photographed under UV illumination. Blue and white triangles point to bands or positions that indicate the genome is circularly permuted and terminally redundant (see legend to right panel, below).

Right Panel: Virtual gel of restriction fragments of ChaoS9 DNA, based on the genome sequence.
Restriction enzymes are shown at the top, and each are suffixed with either -L or -C to indicate bands predicted from linear (-L) or circular (-C) DNA. For these simulations, the starting base used for the linear form of the genome was identified by comparison and alignment of the ChaoS9 genome with those of phiH1 and phiCh1. Size M, represent HindIII-cut lambda DNA size markers, with sizes of fragments indicated at the left edge in kbp.
Blue triangles indicate bands that are expected to be present if the DNA is circularly permuted and terminally redundant. White triangles indicate bands predicted to be under-represented or absent if the DNA is circularly permuted and terminally redundant. These bands would be equimolar with the other fragments if the genome ends were formed by precise cutting.

genes MDPI

Supplementary Table S1. DNA primers used for PCR or sequencing viral DNA[1].

DNA primer	Sequence (5′- 3′)	Position (nt)	Strand/Direction
HPP_001	tgtcctcgtcgtagttgaa	20786-20804	R
HPP_002	agtttcacacgcaagcac	20099-20116	F
HPP_003	cgtctccgagatcaatca	19175-19192	R
HPP_004	gagtgaccgttttccacc	18599-18616	F
HPP_005	tcgttatgtttggttgctg	7771-7789	R
HPP_006	ggacgaacagatcgaagaaa	6808-6827	F
HPP_007	gttgcactcatggtcagg	4079-4096	R
HPP_008	tacctcgaacgcaagctc	3537-3554	F
HPP_009	accggatcttgtagccag	2659-2676	R
HPP_010	cgaaggtctacgagttgtcc	2148-2167	F
HPP_011	gcgtgactacgagttcaca	27132-27150	R
HPP_012	gagacgccttttcgatga	26690-26707	F
HPP_013	gcaccaaccgtatggtc	25949-25965	R
HPP_014	tgcccagctcacgacg	21034-21049	F
HPP_015	atcaggtaacaatcacggcac	24652-24672	F
HPP_016	gatatcgccgtaattcgag	24092-24110	R
HPP_017	catctccatcaccaggttt	4254-4272	R
HPP_018	tacctcgaacgcaagctc	3537-3554	F
HPP_019	ccagtactcgcggatgtt	2571-2588	R
HPP_020	actgtgagcaagggacca	1800-1817	F

[1]nucleotide positions and strand/direction refer to the genome sequence deposited at Genbank under the accession MK310226.

Supplementary Table S2

Table S2. Revisions of phiCh1 genes after re-sequencing the genome[a].

Locus_Tag	ORF[a]	Annotation[b]	revision
PhiCh1_010	ORF2	terminase large subunit TerL	point mutation
PhiCh1_015	-	uncharacterized protein	Frameshift; no gene call in phiCh1
PhiCh1_020	ORF3	-	frameshift; start codon shifted
PhiCh1_035	ORF6	portal protein Por	frameshift; start codon shifted; point mutation
PhiCh1_040	ORF7	head morphogenesis protein gpC	point mutation
PhiCh1_045	ORF8+ORF9	prohead protease gpDB	frameshift; ORF fusion
-	ORF10	-	spurious ORF[c]
PhiCh1_060	ORF13	head-tail adaptor protein Ada	point mutation
PhiCh1_105	ORF22+ORF23	tape-measure tail protein Tpm	frameshift; ORF fusion

PhiCh1_120	ORF26+ORF27	uncharacterized protein	frameshift; ORF fusion
PhiCh1_140	ORF31	uncharacterized protein	start codon shifted
PhiCh1_155	ORF34	repeat-containing tail fiber protein	point mutations
PhiCh1_160	-	uncharacterized protein	missing gene call in phiCh1, corresponds to Nmag_4285
PhiCh1_170	-	uncharacterized protein	missing gene call in phiCh1, corresponds to Nmag_4283
PhiCh1_175	ORF36	repeat-containing tail fiber protein	point mutation
PhiCh1_200	ORF41+ORF42	uncharacterized protein	frameshift; ORF fusion
PhiCh1_230	-	uncharacterized protein	missing gene call in phiCh1, corresponds to Nmag_4297
-	ORF50	-	spurious ORF[c]
-	ORF51	-	spurious ORF[c]
-	ORF52	-	spurious ORF[c]
PhiCh1_245	ORF53+ORF54	plasmid replication protein RepH	frameshift; ORF fusion; 18-bp-deletion
PhiCh1_275	ORF60	uncharacterized protein	frameshift; replacment of C-terminal region; point mutation

PhiCh1_315	ORF68	uncharacterized protein	start codon shifted
PhiCh1_335	ORF72	uncharacterized protein	start codon shifted
PhiCh1_360	ORF77	CxxC motif protein	start codon shifted
PhiCh1_365	ORF78	uncharacterized protein	start codon shifted
PhiCh1_370	ORF79	repressor protein	start codon shifted; silent point mutation
PhiCh1_385	-	CxxC motif protein	missing gene call in phiCh1 and in pNMAG03; homolog of phiH1_415
PhiCh1_415	ORF87	uncharacterized protein	start codon shifted
PhiCh1_435	ORF91	uncharacterized protein	start codon shifted; frameshift in the upstream region which was previously considered coding

[a]ORF, open reading frame numbers, as given in GenBank accession AF440695.1

[b]Annotation, as given in the revised GenBank accession (this study) MK450543.

[c] A spurious ORF is an open reading frame which is considered not to code for a protein.

Supplementary Table S3

Table S3. CRISPR spacers matching ChaoS9.

No.	[1]CRISPR spacer matches to ChaoS9	spacer origin	ChaoS9 target locus_tag (gene)
1	DR: GCTTCAACCGCACAAGCGTTCGTCTGAAAC (*Hfx. volcanii*)<br>ChaoS9 82 ACGAAACAACTCAACCTCATGGGAGCACCAGAC 114<br>Ga0136589 1 ACGAAACAACTCAACCTCATGGGAGCACCAGAC 33<br>*********************************	Antarctic Rauer Lake Metagenome Filla 3 #764;<br>3300012033:Ga0136589_1233048:1:1	Intergenic, just downstream of *terS*
2	DR: GTTTCAGACGAACGCTCGTGCGGTTGAAGC (*Har. hispanica*)<br>ChaoS9 89 AACTCAACCTCATGGGAGCACCAGACGAGTTCCAT 123<br>Ga0136562 1 AACTCAACCTCATGGGAGCACCAGACGAGTTTCAT 35<br>******************************* ***	Antarctic Club Lake Metagenome #312;<br>3300012108:Ga0136562_137316:1:134	Intergenic, just downstream of *terS*
3	DR: GTTTCAGACGTACCCTCGTGGGGTTGAAGT (*Halobacterium* sp. DL1)<br>ChaoS9 589 GTGAATCACGAGGGGCTCGAGGACGCCTTCATGTC 623<br>Ga0075105 35 GTGAACCACGAGGGGCTCGAGGACGCCTTCATGTC 1<br>***** *****************************	Antarctic Deep Lake Metagenome 02WF5;<br>3300005928:Ga0075105_1087484:1:204	HTH-domain protein
4	DR: ACTTCAACCCCACGAGGGTACGTCTGAAAC (*Halobacterium* sp. DL1)<br>ChaoS9 23650 TCGCCGCGACCGACGTTGCCCTACCGACAGTTGTC 23684<br>Ga0136556 1 TCGCTGCGACCGACGTTGCCCTACCGACAGTTGTC 35<br>**** ******************************	Antarctic Rauer Lake Metagenome Torckler E6 #831;<br>3300012182:Ga0136556_1102723:1:498	Tail-fiber gene
5	DR: GCTTCAATCCCTCACAGGTCCGTCTGAAAC (haloarchaeon HSR6)<br>ChaoS9 28659 CGCGTCCATCGCCGTCTGGGAGGTGCTCACCGCGT 28693<br>Ga0136590 35 CGCGTCCATCGCCGTCTGGGAGGTGCTCACCGCGT 1<br>***********************************	Antarctic Rauer Lake Metagenome Filla 3 #765;<br>3300012268:Ga0136590_1135058:1:103	Tail-fiber gene
6	DR: GTTTCAGACGTACCCTCGTGGGGTTGAAGC (*Halobacterium* sp. DL1)<br>ChaoS9 33294 AGGACGTCGACGACCTCCCGGACGACGTCCCCAC 33327<br>Ga0075134 34 AGGACGTCGACGACCTCCCGGACGACGTCCCCAC 1<br>**********************************	Antarctic Rauer Lake 3 Metagenome Rauer3;<br>3300005926:Ga0075134_140743:1:74	Hypothetical protein
7	DR: GTTTCAGACGCACCCTTGTGGGGTTGAAGT (haloarchaeon DL31)<br>ChaoS9 33368 TACTACTACTACCAGTGGCGGGAGGGCAGCAAGGT 33402<br>Ga0136565 35 TACTACTACTACCAGTGGCGGGAGGGCAGCAAGGT 1<br>***********************************	Antarctic Deep Lake Metagenome #1;<br>3300012121:Ga0136565_1012662:1:657	Hypothetical protein
8	DR: ACTTCAACCCCACGAGGGTACGTCTGAAAC (*Halobacterium* sp. DL1)<br>ChaoS9 33686 GCCTTCCCGGACAGCCGCGGGCTCTTCTGCGAGG 33719<br>Ga0075133 1 GCCTTCCCGGACAGCCGCGGGCTCTTCTGCGAGG 34	Antarctic Rauer Lake 1 Metagenome Rauer1;<br>3300005925:Ga0075133_118741:1:5	*(int2)*

	`**********************************`		
9	DR: GTCGCGACGCCGTAGAAACCCGCCCTGGGATTGAAAC (*Haloarcula* sp.)<br>ChaoS9 33825 GGCTCCGGTCGTTCGGGAAGTCCTTCTGGATGGACGTCG 33863<br>Ga0136575 1 GGCTCCGGTCGTTCGGGAAGTCCTTCTGGATGGCCGTCG 39<br>`********************************* *****`	Antarctic Deep Lake Metagenome #136;<br>3300012106:Ga0136575_136552:1:151	(*int2*)
10	DR: GTTTCAGACGGACCTGTGAGGGATTGAAGC (haloarchaeon HSR6)<br>ChaoS9 35871 GTCGCCTCGCTCGTTTCACTCGTTTCACTCATGC 35904<br>Ga0136589 2 GTCGCCTCGCTCGTTTCACTCGTTTCACTCATGC 35<br>`**********************************`	Antarctic Rauer Lake Metagenome Filla 3 #764;<br>3300012033:Ga0136589_1254563:1:95	(*parA*)
11	DR: CTTCAACCCCACGAGGGTACGTCTGAAAC (*Halobacterium* sp. DL1)<br>ChaoS9 40040 CTTGTGGTCGGGGACGTCATCGGGCGCGATGAGGTC 40075<br>Sbjct 36 CTTGTGGTCGGGGACGTCATCGGGCGCGATGAGGTC 1<br>`************************************`	Antarctic Deep Lake TFF Metagenome 2006 #1;<br>3300012127:Ga0136608_1093222:1:60	Hypothetical protein
12	DR: GCTTCAACCCCACAAGGGTTCGTCTGAAAC (*Hfx. volcanii)*<br>ChaoS9 40545 GCCCCGTGTCTCTCTCCACCGGTGCTCGTTCTGCTC 40580<br>G15300SP7336 1 GCCCCGTGTCTCTCTCCACCGGTGCTCGTTCTGCTC 36<br>`************************************`	Lake Meyghan, Iran; metagenome	CxxC domain protein
13	DR: GTTTCAGACGTACCCTTGTGGGGTTGAAG (haloarchaeon DL31)<br>ChaoS9 40915 GAGCGGGCCCTGGACGGAGTCGAGGTTCTCCTCGATG 40951<br>Ga0136556 37 GAGCGGGCCCTGGACGGAGTCGAGGTTCTCCTCGATG 1<br>`*************************************`	Antarctic Rauer Lake Metagenome, Torckler E6 #831;<br>3300012182:Ga0136556_1061549:1:553	Hypothetical protein
14	DR: CAGGACAGTAGAAACCCAGAACGGGATTGAAAC (haloarchaeon DL31)<br>ChaoS9 41002 CGCCTCCAGGACGACGCCGGTCGCGACGGTCTCGCA 41037<br>Ga0136575 1 CGCCTCCAGGACGACGCCGGTCGCGACGGTCTCGCA 36<br>`************************************`	Antarctic Deep Lake Metagenome #136;<br>3300012106:Ga0136575_145068:1:106	Hypothetical protein
15	DR: GTTTCAGACGTACCCTCGTGGGGTTGAAGT (*Halobacterium* sp. DL1)<br>ChaoS9 41018 CCGGTCGCGACGGTCTCGCACATCGTGTGGATCCAGTT 41055<br>Ga0136603 38 CCGGTCGCGACGGTCTCGCACATCGTGTGGACCCAGTT 1<br>`******************************* ******`	Antarctic Deep Lake TFF Metagenome #695;<br>3300012265:Ga0136603_1006046:1:199	Hypothetical protein
16	DR: GCTTCAAACCCACGAGGGGTTCGGCTGAAAG (*Hrr. lacusprofundi*)<br>ChaoS9 43406 GACGACCACGCCTACCTCGTCATCGAGACCGAGGT 43440<br>Ga0075134 1 GACGACCACGCCTACCTCGTCATCGAGACCGAGGT 35<br>`***********************************`	Antarctic Rauer Lake 3 Metagenome Rauer3;<br>3300005926:Ga0075134_103117:1:753	Hypothetical protein
17	DR: GTTTCAGACGAACGCTCGTGCGGTTGAAGCA (*Har. hispanica*)<br>ChaoS9 43763 CGGCCGAGGTCGAGGAGACGCTCGCCGGCGACGCGA 43798<br>Ga0136556 36 CGGCCGACGTCGAGGAGACGCTCGCCGGCGACGCGA 1<br>`******* ****************************`	Antarctic Rauer Lake Metagenome Torckler E6 #831;<br>3300012182:Ga0136556_1274466:1:86	Hypothetical protein
18	DR: GTTTCAGACGGACCTGTGAGGGATTGAAGC (haloarchaeon HSR6)<br>ChaoS9 43891 ACGCGCCTCGAGGAGTTCGCGGCCTGGGTGTCGGGC 43926<br>Ga0075134 1 ACCCGCCTCGAGGAGTTCGCGGCCTGGGTGTCGGGC 36<br>`** *********************************`	Antartic Rauer Lake 3 Metagenome Rauer3;<br>3300005926:Ga0075134_104175:1:224	Hypothetical protein
19	DR: CTTTCAGCCGAACCCCTCGTGGGTTTGAAGC (*Hrr. lacusprofundi*)		Hypothetical protein

	ChaoS9 43929 GCTCTGCCTCGACGCCAGCGCCCGGAACGACGACGG 43964<br>Sbjct 36 GCTCTGCCTCGACGCCAGCGCCCGGAACGACGACGG 1<br>************************************	Antarctic Club Lake TFF Metagenome #397;<br>3300012114:Ga0136595_1046963:1:111	
	DR: GTTTCAGACGTACCCTCGTGGGGTTGAAGT (*Halobacterium* sp. DL1)		
20	ChaoS9 46530 CCAGCAGCTGCACGCAGACGGCGAGGTCTACCAGCC 46565<br>CP007061.1 36 CCAGCAGCTCCACGCAGACGGCGAGGTCTACCAGCC 1<br>********* **************************	*Halobacterium* sp. DL1 (CP007061.1)<br>Deep Lake, Antarctica	Hypothetical protein
	DR: GTTGCAACGGGGAGAAAACCCACTACGGGATTGAAAC (*Hqr. walsbyi* C23)		
21	ChaoS9 48364 GGCCCTGGGGGCATCCCCGGTGATGTCCCGATGAGCC 48400<br>G8097SP4086 1 GGCCCTGGGGGCATCCCCGGTGATGTCCCGATGAGCC 37<br>*************************************	Alviso Ponds, USA; metagenome	Hypothetical protein
	DR: GTTTCAGACGGACCCTCGTGGGGTTGAAGC (*Halorhabdus tiamatea*)		
22	ChaoS9 48423 AGACTGCGGCACGCCCTGGCTCATCGAGCGCCAG 48456<br>Ga0102939 1 AGACTGCGGCACGCCCTGGCTCATCGAGCGCCAG 34<br>**********************************	Salt Pond MetaG R2_B_H2O_MG;<br>3300007631:Ga0102939_1208718:1:295	(*repH*)
	DR: GTTTCAGACGTACCCTCGTGGGGTTGAAGT (*Halobacterium* sp. DL1)		
23	ChaoS9 49273 CTGGCGCGCCGGCTCGGCACGCAGCGCGCGCCGGTC 49308<br>Ga0136606 1 CTGGCTCGCCGGCTCGGCACGCAGCGCGCGCCGGTC 36<br>***** ******************************	Antarctic Deep Lake Metagenome #680;<br>3300012262:Ga0136606_1020860:1:315	(*repH*)
	DR: ACTTCAACCCCACGAGGGTACGTCTGAAAC (*Halobacterium* sp. DL1)		
24	ChaoS9 49405 CAGGACCAGAGCCCGGACCAGCGCGAGCGCGTCGTC 49440<br>Ga0075134 36 CAGGACCAGAGCCCGGACCAGCGCGAGCGCGTCGTC 1<br>************************************	Antarctic Rauer Lake 3 Metagenome Rauer3;<br>3300005926:Ga0075134_111160:1:225	(*repH*)
	DR: GCTTCAATCCCTCACAGGTCCGTCTGAAAC (haloarchaeon HSR6)		
25	Query 49536 GACCGCGTTTAACGAACGGTGGACTGGACGGGGAC 49570<br>Ga0136557 1 GACCGCGTTTAACGAACGGTGGACTGGACGGGGAC 35<br>***********************************	Antarctic Rauer Lake Metagenome Torckler E6 #832;<br>3300012178:Ga0136557_1250606:1:11	(*repH*)
	DR: GTTTCAGACGGACCTGTGAGGGATTGAAGC (haloarchaeon HSR6)		
26	ChaoS9 49891 TTTAGCGACTCGTTGCATCCGTCCGACCATGGACGT 49926<br>Ga0136606 36 TTTAGCGACTCGTTGCATCCGTCCGACCATGGACGT 1<br>************************************	Antarctic Deep Lake Metagenome #680;<br>3300012262:Ga0136606_1013502:1:363	(*repH*)
	DR: GTTTCAGACGGACCTGTGAGGGATTGAAGC (haloarchaeon HSR6)		
27	ChaoS9 50224 TGCCTCACGCCGGAACGGCTGGAGTCCGCGGAC 50256<br>Ga0136556 1 TGCCTCACGCCGGAACGGCTGGAGTCCGCGGAC 33<br>*********************************	Antarctic Rauer Lake Metagenome Torckler E6 #831;<br>3300012182:Ga0136556_1267814:1:84	(*repH*)
	DR: GCTTCAATCCCTCACAGGTCCGTCTGAAAC (haloarchaeon HSR6)		
28	ChaoS9 50184 CACTGCCCGGTCGTTAACCCACTGGCGGATCTTCGA 50219<br>Ga0075107 1 CACCGCCCGGTCGTTAACCCACTGGCGGATCTTCGA 36<br>*** ********************************	Antarctic Deep Lake Metagenome 022AM;<br>3300005929:Ga0075107_1103432:1:6	(*repH*)
	DR: GTCGCGACGGGGAGAAAACCCACTGCGGGATTGAAAC (*Haloarcula* sp.)		
29	ChaoS9 50724 GCTGATCGGCTCGTTCGTCCTGGCCGGGAACTTCGG 50759<br>G10SP347 36 GCTGATCGGCTCGTTCGTCCTGGCCGGGAACTTCGG 1<br>************************************	Alviso Ponds, USA; metagenome	(*repH*)

	DR: GTTTCAGACGTACCCTCGTGGGGTTGAAGT (*Halobacterium* sp. DL1)		
30	ChaoS9 50881 GAGTTCGACCGCGAGCAGTTCGCCGTCGCGACGTC 50915<br>Ga0136558 1 GAGTTTGACCGCGAGCAGTTCGCCGTCGCGACGTC 35<br>***** *****************************	Antarctic Rauer Lake Metagenome Torckler E6 #833;<br>3300011189:Ga0136558_1268702:1:253	(*repH*)
	DR: ACTTCAACCCCACAAGGGTGCGTCTGAAAC (haloarchaeon DL31)		
31	ChaoS9 50940 CCGGCCGACCCGGGAGACCGTCTCGCTGCTCCGG 50973<br>Ga0136607 1 CCGGCCGACCCGGGAGACCGTCTCGCTGCTCCGG 34<br>**********************************	Antarctic Deep Lake Metagenome #681;<br>3300011181:Ga0136607_1027372:1:399	(*repH*)
	DR: GTTTCAGACGGACCTGTGAGGGATTGAAGC (haloarchaeon HSR6)		
32	ChaoS9 50956 ACCGTCTCGCTGCTCCGGCTGTTCACCGGGAACCC 50990<br>Ga0075134 35 ACCGTCTCGCTGCTCCGGCTGTTCACCGGGAACCC 1<br>***********************************	Antarctic Rauer Lake 3 Metagenome Rauer3;<br>3300005926:Ga0075134_106034:1:601	(*repH*)
	DR: ACTTCAACCCCACGAGGGTACGTCTGAAAC (*Halobacterium* sp. DL1)		
33	ChaoS9 51038 CTCGCGAACTCCGGCCGAGCGAGGTCCGGTTCGCG 51072<br>Ga0136590 35 CTCGCGAACTCCGGCCGAGCGAGGTCCGGTTCGCG 1<br>***********************************	Antarctic Rauer Lake Metagenome Filla 3 #765;<br>3300012268:Ga0136590_1001959:1:403	(*repH*)
	DR: GTTTCAGACGTACCCTCGTGGGGTTGAAGT (*Halobacterium* sp. DL1)		
34	ChaoS9 51101 TCCCGCACTGCTCGCCGGGCGGTCGGGCTATCGTGA 51136<br>CP007061.1 1 TCCCGCACTGCTCGCCGGGAGGCCGGGCTATCGTGA 36<br>******************* ** *************	*Halobacterium* sp. DL1 (CP007061.1)<br>Deep Lake, Antarctica	(*repH*)
	DR: GCTTCAATCCCTCACAGGTCCGTCTGAAAC (haloarchaeon HSR6)		
35	ChaoS9 51328 GAGATCGTGCCGTGGCCCGTCGAGGACGCCGA 51359<br>Ga0075133 32 GAGATCGTGCCGTGGCCCGTCGAGGACGCCGA 1<br>********************************	Antarctic Rauer Lake 1 Metagenome Rauer1;<br>3300005925:Ga0075133_103592:1:1140	(*repH*)
	DR: GTTTCAGACGTACCCTCGTGGGGTTGAAGC (*Halobacterium* sp. DL1)		
36	ChaoS9 51979 GCTCTCAAGCCGCCGAGAGGTGGTGTCCTGCTCAG 52013<br>Ga0136591 1 GCTCTCAAGCCGCCGAGAGGTGGTGTCCTGCTCAG 35<br>***********************************	Antarctic Rauer Lake Metagenome Filla 3 #767;<br>3300012267:Ga0136591_1130480:1:202	CxxC domain protein
	DR: ACTTCAACCCCACAAGGGTGCGTCTGAAAC (*Halobacterium* sp. DL31)		
37	ChaoS9 52454 GACTGGGTCCATCTCGTCGAGCGCGTCCAGGAGCT 52488<br>Ga0075106 1 GACTGGGTCCATCTCGTCGAGCGCGTCCAGGAGCT 35<br>***********************************	Antarctic Deep Lake Metagenome 02WF4;<br>3300005930:Ga0075106_1079420:1:90	CxxC domain protein
	DR: GTTTCAGACGTACCCTTGTGGGGTTGAAGC (*Halobacterium* sp. DL31)		
38	ChaoS9 52880 AGCTGATCACCACCAACGCGCCAGGACCACGGCGA 52914<br>Ga0136606 1 AGCTGATCACCACCAACGCGCCAGGACCACGGCGA 35<br>***********************************	Antarctic Deep Lake Metagenome #680;<br>3300012262:Ga0136606_1075654:1:136	Intergenic?
	DR: GTCGCGACGGGGAGAAAACCCACTGCGGGATTGAAAC (*Haloarcula* sp.)		
39	ChaoS9 54513 CGTCAACGGCGAGGACGAGTCGTTCGGCGACACGAT 54548<br>G10SP3266 36 CGTCAACCGCGAGGACGAGTCGTTCGGCGACACGAT 1<br>******* ****************************	Alviso Ponds, USA; metagenome	DUF271 domain protein

[1] The matching spacer sequences were found in the following NCBI bioprojects using the crass program: PRJNA337743 (SRA SRR4030040; Alviso Ponds, San Francisco, CA, USA, metagenome)[1]; PRJEB18068 (Lake Meyghan, Iran; metagenome). Spacers from Antarctic lake metagenomes and salt pond MetaG were found by spacer BLASTn searches at the Integrated Microbial Genomes/Virus (IMG/VR) webserver (https://img.jgi.doe.gov). Aligned sequences show nt positions for ChaoS9, and asterisks indicated identical bases. DR, direct repeat sequence. The bracketed species at the right of DR sequences show haloarchaea with the most closely similar DR sequences, as provided by searches at CrisprFinder (http://crispr.i2bc.paris-saclay.fr/crispr/). Studies reporting the Lake Meyghan (Iran) metagenome have been published by [2]; the antarctic lake metagenomes by [3], and the Alviso Ponds, San Francisco (USA) metagenome by [1]. The MetaG R2_B_H2O_MG metagenome is part of a study of salt pond microbial communities from South San Francisco (USA), GOLD project accession Gp0125935.

Supplementary Table S4

Table S4. CRISPR spacers matching phiH1 and phiCh1.

No.	[1]CRISPR spacer matches to phiH1/phiCh1	spacer origin	Target locus_tag (gene)
1	phiH1 24561 ACGCGGTGAGCACCTCCCAGACGGCGATGGACGCG 24595<br>Ga0136590 1 ACGCGGTGAGCACCTCCCAGACGGCGATGGACGCG 35<br>***********************************	Antarctic Rauer Lake Metagenome<br>3300012268:Ga0136590_1135058:1:103	PhiH1_165 (Repeat-containing tail fiber)
2	phiH1 24561 ACGCGGTGAGCACCTCCCAGACGGCGATGGACGCG 24595<br>Ga0075105 35 ACGCGGTGAGCACCTCCCAGACGGCGATGGACGCG 1<br>***********************************	Antarctic Deep Lake Metagenome<br>3300005928:Ga0075105_1094377:1:97	PhiH1_165 (Repeat-containing tail fiber)
3	phiH1 24575 TCCCAGACGGCGATGGACGCGGTGAGCACGTC 24606<br>Ga0075130 39 TCCCAGACGGCGATGGACGCGGTGAGCACGTC 8<br>********************************	Antarctic Deep Lake Metagenome<br>3300005927:Ga0075130_171271:1:35	PhiH1_165 (Repeat-containing tail fiber)
4	phiCh1 6319 GAGATCTCCGACGTCGACATCCCGGCGGT 6347<br>Ga0075133 2 GAGATCTCCGACGTCGACATCCCGGCGGT 30<br>*****************************	Antarctic Rauer Lake Metagenome<br>3300005925:Ga0075133_107083:1:157	PhiCh1_045 (prohead protease gpB)
5	phiCh1 23738 TCCCAGACGGCGATGGACGCGGTGAGCGACTCCCA 23772<br>ADL13m1u 35 TCCCAGACGGCGATGGACGCGGTGAGCGCTTCCCA 1<br>**************************** *****	Antarctic Deep Lake Metagenome<br>2100351014:ADL13m1u_GQIGRQ001CCDN3:1	PhiCh1_155 (repeat-containing tail fiber)

[1] Spacers were found by spacer BLASTn searches at the Integrated Microbial Genomes/Virus (IMG/VR) webserver (https://img.jgi.doe.gov; accessed 20 December 2018). Aligned sequences show nt positions for the virus genome, and asterisks indicate identical bases.
